# Genome-wide standing variation facilitates long-term response to bidirectional selection for antibody response in chickens

**DOI:** 10.1186/s12864-016-3414-7

**Published:** 2017-01-18

**Authors:** Mette Lillie, Zheya Sheng, Christa F. Honaker, Ben J. Dorshorst, Christopher M. Ashwell, Paul B. Siegel, Örjan Carlborg

**Affiliations:** 1 Department of Medical Biochemistry and Microbiology, Genomics, Uppsala University, Uppsala, 75123 Sweden; 2 Key Laboratory of Agricultural Animal Genetics, Breeding and Reproduction of Ministry of Education, College of Animal Science and Technology, Huazhong Agricultural University, Wuhan, 430070 People’s Republic of China; 3 Department of Animal and Poultry Sciences, Virginia Polytechnic Institute and State University, Blacksburg, VA 24061 USA; 4 Prestage Department of Poultry Science, North Carolina State University, Raleigh, NC 27695 USA

**Keywords:** Pooled genome sequencing, Selective sweeps, Virginia chicken lines, Sheep red blood cells, Antibody response

## Abstract

**Background:**

Long-term selection experiments provide a powerful approach to gain empirical insights into adaptation, allowing researchers to uncover the targets of selection and infer their contributions to the mode and tempo of adaptation. Here we implement a pooled genome re-sequencing approach to investigate the consequences of 39 generations of bidirectional selection in White Leghorn chickens on a humoral immune trait: antibody response to sheep red blood cells.

**Results:**

We observed wide genome involvement in response to this selection regime. Many genomic regions were highly differentiated resulting from this experimental selection regime, an involvement of up to 20% of the chicken genome (208.8 Mb). While genetic drift has certainly contributed to this, we implement gene ontology, association analysis and population simulations to increase our confidence in candidate selective sweeps. Three strong candidate genes, *MHC*, *SEMA5A* and *TGFBR2*, are also presented.

**Conclusions:**

The extensive genomic changes highlight the polygenic genetic architecture of antibody response in these chicken populations, which are derived from a common founder population, demonstrating the extent of standing immunogenetic variation available at the onset of selection.

**Electronic supplementary material:**

The online version of this article (doi:10.1186/s12864-016-3414-7) contains supplementary material, which is available to authorized users.

## Background

A key aim in the study of evolutionary genetics is to identify loci that facilitate adaptation. The importance of de novo mutations and standing genetic variation to the mode and tempo of the adaptive process has been debated, as well as the importance of fixation for adaptation [1–3]. Through necessity, de novo mutations are essential within bacterial systems, where beneficial mutations arising within clonal populations will sweep to fixation. Interaction and competition between de novo mutations observed in experimental populations have provided valuable insights into the process of adaptation within microbial species [4–6]. Research in sexual organisms, however, reveals that selection on standing genetic variation is the predominant basis of adaptation in higher eukaryotes [7–10]. In this case, standing genetic variants are present in the population at low frequencies, maintained by neutral or slightly negative selection. Within an altered environment, these standing variants will gain a selective advantage and increase in frequency in the population to reach fixation. Depending on the genetic architecture of functional traits and population structure, adaptation can also be facilitated by moderate allele frequency differences at multiple genes, without producing such dramatic sweep-to-fixation signatures [1, 2, 11].

The number of genes underlying an adaptive process often belies the complexity of the selective environment. For traits with a complex genetic basis, such as aging and courtship, experimental evolution studies in *Drosophila* have demonstrated genome-wide involvement in response to selection [12, 13]. For immune traits, the extent of genome involvement in the adaptive response can vary. For example, in *Drosophila melanogaster*, nearly 5% of the genome, and 42 genes, were suggested to be involved in an 150% increase in parasitoid resistance [14], whereas only a few genes were identified and functionally validated as the targets of selection for resistance to *Drosophila* C virus [15].

**Fig. 1:**
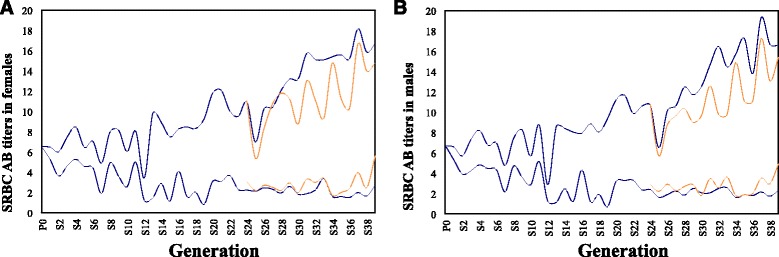
Changes in mean sheep red blood cell (SRBC) antibody (AB) titers across 39 generations of selection in Virginia AB chicken lines for females (**a**) and males (**b**). Selected lines shown in *blue*, with relaxed lines shown in *yellow*

Here we use a genomic approach to investigate the consequences of long-term, bidirectional selection on a single immune trait from a base population of randombred White Leghorn chickens [16]. In brief, selection was performed for high (HAS) or low (LAS) day 5 antibody production to an intravenous challenge of sheep red blood cells (SRBC) (further details can be found in [16–18]). At generation 39, the HAS and LAS lines showed an average 6.5 fold difference in antibody titers (Fig. 1). Pooled genome sequencing was carried out for each selected line at generation 39 (HAS39 and LAS39) allowing the identification of regions of high differentiation (*F*_*ST*_). Due to the bidirectional selection regime, the identified selection signatures would result primarily from selection on standing genetic variants [10, 19]. Relaxed lines (random-mated sublines) founded for both lines at generation 24 were also genome sequenced in pools at generation 16 (HAR16 and LAR16). The genetic effects of the sweeps were estimated using data from an F_2_ intercross between the HAS and LAS lines at generation 32.

We observed over 200 regions across the genome affected by the selection regime, characterized by spans of extreme differentiation between HAS39 and LAS39. These would include true selective sweeps and those regions displaying this pattern due to the random genetic drift experienced by the small selected populations. To identify strong candidate sweeps from this large set of differentiated regions, we overlapped regions with associations detected to antibody response in an F_2_ intercross between the selected lines, used enrichment analysis to detect regions with immune genes and identified overlaps with immune associations mapped in other studies/populations. In this way, a high-confidence set of candidate selective sweep regions may be inferred. Three particularly relevant candidate genes, *MHC*, *SEMA5A* and *TGFBR2*, are presented in more detail.

## Results

### A large genome-wide footprint of selection in the divergently selected lines

A total of 711,149 1000 bp windows were analyzed in *Popoolation2*. A summary of the population-statistics for the four analyzed populations is provided in Table 1. A general reduction in heterozygosity in these regions was observed relative to genome-wide heterozygosity in the selected lines. Differentiation (*F*_*ST*_) was also greater between the S39 than the R16 lines.

**Table 1:** Summary statistics on pooled genomes of Antibody line populations

	HAS39	LAS39	HAR16	LAR16
Sweep heterozygosity	0.120	0.127	0.191	0.156
Genome-wide heterozygosity	0.199	0.190	0.204	0.166
Sweep Het/Genome-wide Het	0.603	0.666	0.939	0.940
*F*_*ST*_	0.519		0.323	

*F*_*ST*_ from population comparisons between HAS39 with LAS39, and between HAR16 and LAR16

**Fig. 2:**
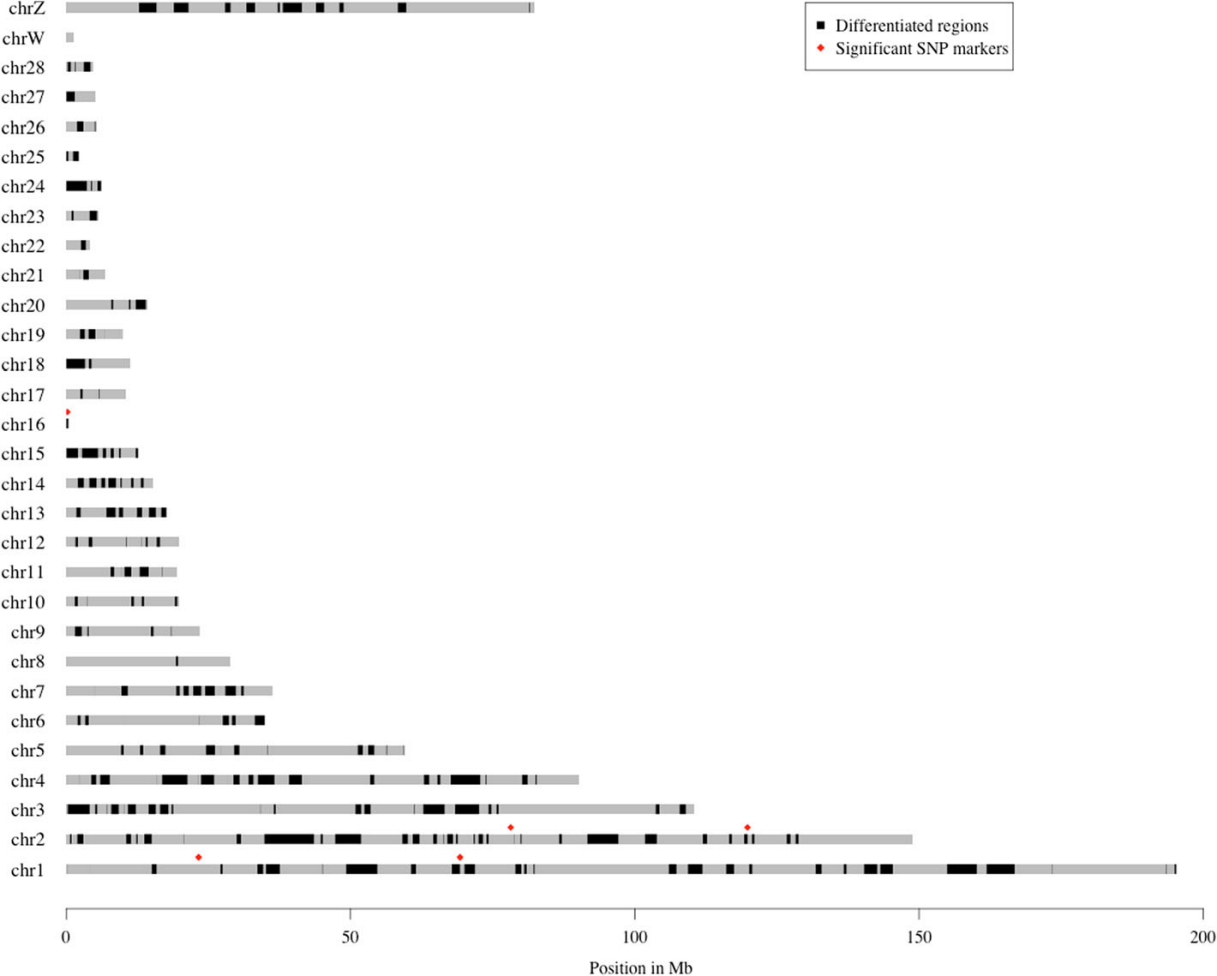
Locations of highly differentiated genomic regions (*black*) across the chicken chromosomes. The SNP markers associated with 5-day antibody titer in our backward-elimination based analysis of the F2 intercross between HAS32 and LAS32 are indicated with *red* diamonds

After clustering of windows located less than 0.5 Mb apart, and removing sweep-regions with a single 1000 bp window or only 2 SNPs, 224 highly differentiated regions were retained (Fig. 2; Table listing differentiated regions in Additional file 1). These regions were located on 50 genome contigs, with 203 across the 29 assembled chromosomes and 1 region on each of 21 unmapped genome scaffolds, spanning a total of 208.8 Mb (20.1% of the assembled galgal4 chicken genome). The regions ranged in length from 1.5 kb to 8.7 Mb (mean/median length: 932/538 kb).

### Estimating the contribution of drift to the allelic divergence between populations

Simulations in SFS_CODE [20] were used to estimate the contribution of genetic drift to the genome-wide divergence between the HAS and LAS lines, an effect that would confound true sweep signatures across these genomes. Simulations were conducted for macro- and micro-chromosome recombination rates (estimated at 2.8 and 6.4 cM/Mb respectively; [21]) and regions of differentiation due to neutral processes are summarized in Table 2. Median lengths were 75,000 and 71,750 bp, respectively, with maximum lengths at 709,000 and 662,500 bp. From 10.8 to 20.3% of the simulated DNA fragments showed stretches of differentiation, emphasising the influence of genetic drift in the selected chicken populations. Quantifying the regions that have differentiated as a result of selection versus drift is impossible, but by overlapping the genomic results with those of other studies, association analysis and investigating deeper into candidate genes, we build confidence that many regions have contributed to the divergence in antibody response observed in the Antibody lines.

**Table 2:** Summary information from simulations

	Micro-chromosomes	Macro-chromosomes
Median length	71,750	75,000
Median *F*_*ST*_	0.366	0.362
Max. length	709,000	662,500
Mean length	109,021	129,536
Fixed %	20.3	10.8

### Many candidate selective sweep regions contain genes related with immune-function

The 224 highly differentiated regions contained a total 3511 annotated genes. To identify potential candidate adaptive genes, as well as to indicate individual candidate sweeps that are more likely to have reached high frequency by selection rather than drift, we subjected these to a Gene Ontology (GO) analysis. Using the DAVID GO analysis, 67 gene IDs were reported to be associated with immune system processes (listed in Additional file 2), and these mapped to 46 regions (also refer to Additional file 1). The PANTHER GO analysis identified 155 gene IDs associated with immune function (listed in Additional file 3) mapping to 70 regions (also refer to Additional file 1). Combining the results from both methods revealed a total of 82 regions overlapping genes associated with immune function. Between 1 and 13 immune genes were found within these regions, with the most observed on GGA 16, notably the Major Histocompatibility Complex (*MHC*) region.

### Several candidate selective-sweep regions overlap with genomic regions associated with immunological traits in chicken

In total, 23 highly differentiated regions overlapped with regions previously reported to be associated with immunological traits in chickens. These included antibody response to SRBC and *Brucella abortus* in Leghorn hens [22], primary and secondary antibody response to SRBC in ISA Warren layer hens [23], innate immunity in layer hens [24], innate and adaptive immunity in layer hens [25], multiple immune traits in the Chinese indigenous breed Bejing-You chicken [26], and differential expression between high and low SRBC antibody responses in White Leghorn females [27] (also refer to Additional file 1).

### Several candidate selective-sweep regions are associated with day 5 antibody titers in an F_2_ intercross between chickens from HAS32 and LAS32

We reanalyzed a previously generated dataset from an F_2_ intercross from generation 32 of the divergent lines. In total, 150 of the 1024 polymorphic markers were highly differentiated, with an allele-frequency difference between HAS32 and LAS32 > 0.7 (SNP markers and locations listed in Additional file 4). This SNP subset was clustered into 63 regions on 24 chromosomes from which a subset of 63 representative SNP markers (1 per region) was selected using a per region backward elimination analysis. These markers were then fitted jointly in a whole-genome multi-locus, backward elimination analysis to identify five SNP markers associated with the selected trait at 20% FDR threshold. Four of these markers were retained in the model at a 5% FDR threshold (Table 3). The identified markers were located on chromosomes 1, 2, and 16 (Fig. 2).

**Table 3:** Genetic effects of SNP markers associated with log_2_(day 5 antibody titers) at a 20% FDR threshold in the F_2_ intercross of Virginia Antibody lines

Marker	Chromosome^a^	Position^b^ (bp)	Estimate^c^ ± std. err.	*P*-value^d^	Sign.^e^
rs14799859	GGA1	23,275,033	1.7 ± 0.5	0.0017	5%
rs15307852	GGA1	69,259,824	1.5 ± 0.5	0.0019	5%
rs14207559	GGA2	78,180,370	2.1 ± 0.4	3.6 × 10^−6^	5%
rs14242328	GGA2	119,824,660	1.6 ± 0.4	0.0006	5%
rs14096690	GGA16	192,620	1.2 ± 0.4	0.0050	20%

^a^ *GGA Gallus gallus* autosome

^b^November 2011 (galGal4) assembly

^c^Additive genetic effect in model including all tabulated loci

^d^Significance for additive genetic effect in model including all tabulated loci

^e^Significance thresholds 5/20% FDR based on which markers were selected

As the linkage disequilibrium is extensive in the F_2_ population, the functional polymorphisms causing the marker-trait associations could located several Mb away from the tested markers. The marker with the most significant association, rs14207559, is located close to a candidate sweep region at GGA2: 78,748,000–78,800,500, which contains the candidate immune gene, semaphorin 5A *SEMA5A* (see below section). Among the markers selected by the 5% FDR threshold, rs14799859 is located on GGA1 between a sweep region ending at 15,887,000 and another beginning at 27,122,500, marker rs15307852 is located just outside the sweep region at GGA1: 67,820,500–69,251,000, and marker rs14242328 falls within the sweep region on GGA2: 119,248,000–119,841,000, none of which contains annotated immune genes. The marker rs14096690 showed only 20% FDR significance in the model, but is located within the sweep region GGA16: 2000–323,000 and notably within the candidate *MHC* region (see below section).

### SEMA5A is a candidate adaptive gene in a selective sweep region associated with day 5 antibody titers

**Fig. 3:**
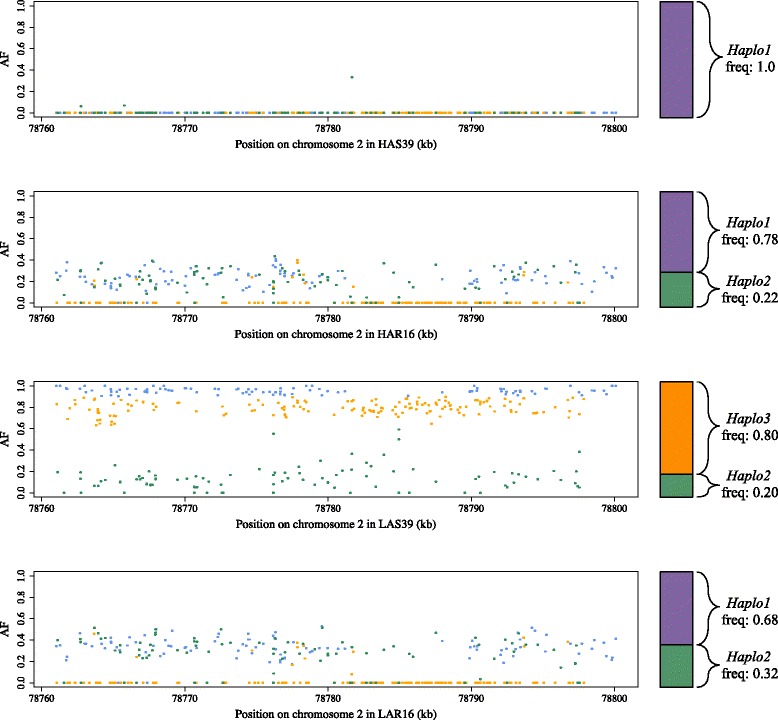
Inferred major SEMA5A haplotypes in HAS39 (*upper*), HAS16 (*middle upper*), LAS39 (*middle lower*), LAR16 (*lower*). Positions are colored based on their contribution to different haplotypes: allele frequencies for SNPs in *green* contribute to Haplo2 and *orange* contribute to Haplo3. SNPs in *blue* contribute to both Haplo2 and Haplo3, so where both of these haplotypes are present, *blue* positions will have an allele frequency approximately equal to 1 as in LAS39 where only one haplotype is present. In the case of LAR16 and HAR16, the *blue* positions will have an allele frequency approximate to Haplo2

The SNP marker rs14207559 that is significantly associated with day 5 antibody titers is located only 568 kb away from, and closely linked to in the F_2_ population, the candidate sweep region GGA2: 78,748,000–78,800,500 (Additional file 5). It is fixed for T in HAS39, while it segregates for T/G in LAS at differentiated frequencies (0.27/0.73: T/G, Table 4). We explored the haplotype-structure of this candidate sweep region in the four analyzed populations in greater detail where the sweep region overlaps with the *SEMA5A* candidate gene (GGA2: 78,760,000–78,800,000 bp; Fig. 3). In this region, 345 positions are fixed in HAS39, but continue to segregate in LAS39 (Additional file 5). These positions in LAS39 contribute towards two major haplotypes, *Haplo2* and *Haplo3*, which are not present in HAS39, and segregate at the approximate haplotype frequencies of 0.2 and 0.8, respectively (Table 5, Fig. 3). When compared to the relaxed lines, both HAR16 and LAR16 have *Haplo1* and *Haplo2* segregating at intermediate frequencies, and lack the *Haplo3* haplotype (Table 5, Fig. 3). (It should be noted that with their smaller sample sizes, haplotype frequencies in the relaxed pooled genomes should be treated with caution and only serve as indications of the allele / haplotype frequencies of the sampled populations. Nevertheless, they are useful to reveal general trends across pooled genome comparisons.)

**Table 4:** Estimates of mean phenotypes for the three genotypes at the significantly associated SNP near *SEMA5A* (rs14207559; GGA2: 78,180,370) in the F_2_ intercross between HAS32 and LAS32

Genotype (rs14207559)	Mean antibody titer	SE
G/G	2.2	0.7
G/T	4.8	0.5
T/T	6.5	0.5

**Table 5:** Allele and haplotype frequencies of the SNP marker rs14207559 and the *SEMA5A* gene-region

	Allele frequency (G; rs14207559)	SEMA5A		
		Haplotype frequencies		
		Haplo1	Haplo2	Haplo3
HAS39	0	1	0	0
HAR16	0	0.78	0.22	0
LAS39	0.732	0	0.2	0.8
LAR16	NA	0.68	0.32	0

The associated SNP marker rs14207559 appears to tag these major haplotypes at this candidate sweep region in the F_2_ population, with the G-allele tagging *Haplo3*, the dominant haplotype in LAS39, and the T-allele tagging both *Haplo1* and *Haplo2* (Table 5, Fig. 3). From these inferred haplotype frequencies, it appears that *Haplo3* has a selective benefit only in LAS and otherwise would be deleterious, thus explaining why it was purged in the LAR line after the relaxation of selection. Pairwise differentiation of this region reinforces this hypothesis, as LAS39 is highly differentiated from HAS39, HAR16, and LAR16, while LAR16 appears less differentiated from HAS39 and HAR16 than LAS39 (Table 6). The two differentiated variants within *SEMA5A* exons are synonymous, with the majority of polymorphisms in this region located in intergenic and intronic regions.

**Table 6:** Pairwise differentiation (mean *F*_*ST*_) between the analyzed lineages across the region overlapping the *SEMA5A* gene and the candidate selective sweep (GGA2: 78,760,824–78,800,500)

	HAS39	LAS39	HAR16
LAS39	0.595		
HAR16	0.137	0.456	
LAR16	0.202	0.438	0.029

### Deletion in *TGFBR2* located in the longest candidate sweep region

Previously, we observed a correlation between the length of selective sweeps and their contribution to adaptation in a similar long-term, single-trait selection-experiment for 8-week body weight in chickens [10]. Therefore, here we investigated the longest candidate sweep region (GGA2: 34,856,000–43,580,000; 8.7 Mb) in more detail. In this region, HAS39 and LAS39 appear fixed for alternative haplotypes across this region, as are their respective relaxed lines. This pattern of fixation indicates that this region was fixed for alternative alleles within the selected lines prior to generation 24, which was the founding generation of the relaxed lines. Such rapid fixation suggests that this sweep represents selection on a standing variant with strong phenotypic effect. Several genes with roles in the immune system are located within this region, including several cytokines, and we also detected a large structural variant within the transforming growth factor beta-receptor 2 gene, *TGFBR2* (ENSGALG00000011442, GGA2: 40,385,525–40,447,574).

By referring to RNAseq data, we identified 9 exons contributing to the transcription of *TGFBR2* (see also Additional file 6). Exons 2-3 and 4-5 of *TGFBR2* appear to be the result from a historical duplication event. Exons 3 and 5 are identical at the sequence level, while exons 2 and 4 share over 90% sequence identity (intron 2-3 shares > 94% sequence identity with intron 4-5). The haplotype that appears fixed in LAS39 has a large deletion overlapping exons 4-5 of this duplicated region. By comparing de novo assemblies of LAS39 and HAS39 genomes, the deletion was localized to GGA2: 40,414,509–40,418,21 and results in a 3712 bp deletion (see also Additional file 6).

### Major histocompatibility complex

Previous studies have traced the changes in MHC allele segregation between the HAS and LAS lines [28, 29]. As such, we simply aimed to add information from the pooled genome sequencing to these results. We confirmed fixation for MHC *B* locus *B* ^*21*^ haplotype in HAS39 and *B* ^*13*^ in LAS39 (Table 7). Also observed was fixation for *B* ^*13*^ in LAR16, while HAR16 continued to contain both *B* ^*21*^ and *B* ^*13*^ haplotypes segregating at the approximate haplotype frequencies 0.73 and 0.27, respectively (Table 7).

**Table 7:** MHC *B* locus *B* ^*21*^ and *B* ^*13*^ haplotype frequencies for different generations in the Virginia Antibody lines. Note that allele frequencies in HAS10 do not equal 1, as a third *MHC* haplotype *B* ^*31*^ was present at a frequency of 0.05 in this generation [29]

	HA		LA		Citation
Generation	*freq(B* ^*21*^ *)*	*freq(B* ^*13*^ *)*	*freq(B* ^*21*^ *)*	*freq(B* ^*13*^ *)*	
S10	0.80	0.15	0.01	0.99	Martin et al. 1990 [29]
S13	0.99	0.01	0.02	0.98	Martin et al. 1990 [29]
S32	1	0	0	1	Dorshorst et al. 2011 [48]
R16	0.73	0.27	0	1	This study

## Discussion

Long-term, bidirectional experimental selection provides a powerful approach to gain empirical insights to the genetics of complex traits. Within the quantitative genetic framework, research has provided knowledge about relationships between the predicted and realized responses to selection, the boundaries of selection in closed populations due to reduction in selectable genetic variation, and physiological limits for further response [30, 31]. With the advent of large-scale molecular genetics and genomics methods, these highly selected populations also become valuable models for studying how the genomes of populations under intense selection for various traits evolve during adaptation [5, 19, 32, 33].

The Virginia Antibody lines are a unique experimental model-population for studying the genetics of long-term selection for an adaptive immune trait in a higher vertebrate. Using whole-genome re-sequencing of pooled DNA from these divergent lines, we observed wide genome involvement in the long-term response to selection. Over 200 regions, covering nearly 20% of the genome, were clearly differentiated between the lines. Despite the action of genetic drift, many regions were the result of bidirectional selection and have contributed to selection response, as suggested by overlaps with immune genes and marker to immune-trait associations in an intercross between the selected lines as well as other independent populations. These candidate selective sweeps are primarily the result of two key factors: first, the selected variants were present at the onset of selection (i.e. that selection has acted on standing genetic variation in the founding population), and second, that selection acts on these standing variants in opposite directions.

These extensive genome changes in this long-term selection experiment suggest that SRBC antibody response is a highly polygenic trait. The 6.5 fold difference in antibody titers between the divergently selected lines has been facilitated by large differentiation at many loci with standing genetic variants. This finding is consistent with those in the Virginia body weight lines, which has been bidirectionally selected for juvenile body-weight for over 55 generations. There, it has been shown that selection on standing variation in a highly polygenic genetic architecture was the main contributor to the long-term selection response in body weight [10].

Genetic drift in these relatively small breeding populations would have contributed not only to random divergence between the selected lines, but also to the fixation of selective sweeps. Previous studies where *Drosophila melanogaster* and *Saccharomyces cerevisiae* were used to model adaptation in vertebrate populations have rarely detected complete selective sweeps [6, 9]. Further, in human studies, the polygenic architecture of most traits disperses the influence of selection and hence adaptation is facilitated by moderate allele frequency changes of multiple loci without dramatic fixation events [2, 34]. In contrast, within our small population, there was loss of heterozygosity within selective sweep regions attributable to fixation of genetic variants. The combination of selection and genetic drift within the Antibody lines have lead to a higher level of fixation of selective sweeps than observed in other model systems.

Antibody production is a major defense mechanism of the humoral immune response. It involves a variety of different cell types with specific functions, including immunological cascades involving the recognition of the foreign cells by T and B immune cells, activation and differentiation of B cells into plasma cells and secretion of specific antibodies [35]. Moreover, this cascade relies on the careful coordination of gene expression through signaling molecules and appropriate receptors, within which there is potential for many genes and genetic variants to have an effect. Our results suggest that not just that many loci play a role in SRBC antibody production, but also that the founder population harbored substantial genetic variation upon which selection could act. This supports the expectation that adaptive immune traits are genetically complex and further emphasizes the need to consider more than just a few key immune genes when studying immunogenetic selection and adaptation even in populations originating from a narrow genetic basis.

Earlier works with the Virginia Antibody lines have, however, shown that the level of antibody-response does not have a direct connection to a general greater immunological efficacy. For example, when the lines selected for high (HAS) and low (LAS) antibody production were faced with different immunological challenges, the HAS were less susceptible to the northern fowl mite (*Ornithonyssus sylviarum*) and Newcastle Disease, but more susceptible to the bacterial pathogens *Escherichia coli* and *Staphylococcus aureus* when compared to LAS [36]. These immunological trade-offs further emphasize the complexity of the immune system, and show fixation of mutations within this selected population that would be otherwise deleterious. Selection for different arms of the immune system resulting in difference in disease resistances have been well characterized in other chicken studies, such as the IAH Compton lines, where different inbred lines show variation in disease susceptibility/ resistance [37], as well as the notable differences in disease resistances observed between broiler and layer chicken breeds [38, 39]. They also serve as a reminder that this study does not provide insights to how and whether breeding can be used to improve the general performance of the immune system, but rather that the immune system of a particular population is likely to be very adaptable when challenged with various disease challenges and that such responses are likely due to selection on available variants in many genes. It is no doubt advantageous for a population to have such variation in host-pathogen interactions to respond to immunological challenges.

We observed many genes with immunological function underlying our candidate sweep regions, while many still did not. Particularly from our association analysis, we found two SNP markers with associations to day 5 antibody titers and closely located candidate immune genes, while the remaining three SNP markers were not located near immune genes involved in selective sweeps. This may be a consequence of our strict GO search for genes with immune function, which would not identify functional genes contributing to the divergent antibody response but without GO immune function terms. Alternatively, genes within the chicken genome lacking annotation in galgal4 would not be identified by our methodology. It should be noted that while our analysis of this previously published dataset focused on the most informative SNP markers (those with allele frequency differences between the lines), this restricted our genome coverage and would have limited the opportunities of identifying associations to day 5 antibody titers.

In the remaining sections, we discuss a number of particularly promising candidate functional genes detected in the identified sweep regions.

### Semaphorin-5A

A sweep region located near the SNP marker (rs14207559) with the strongest association to day 5 antibody titers in the F_2_ intercross between the HAS and LAS lines in generation 32 overlaps the first 7 of 18 exons (39,676 of 136,181 bp) of Semaphorin-5A (*SEMA5A*, ENSGALG00000013051). Semaphorins are a large family of secreted and membrane bound proteins, originally discovered in the nervous system with a role in axon guidance (summarized in [40]). *SEMA5A* is a secreted semaphorin involved in immune regulation and the pathogenesis of autoimmune diseases [40]. It has been implicated in the pathogenesis of immunological diseases such as mastitis in dairy cattle [41], chronic immune thrombocotypenia in humans [40], and neurodevelopmental disorders such as autism in humans [42]. As an immunoregulator, *SEMA5A* influences the expression of cytokines such as IFN-gamma, TNF-gamma, and interleukins [40].

In the Virginia Antibody lines, the sweep region overlapping the *SEMA5A* gene is present in at least three different haplotypes: the major haplotypes referred to here as *Haplo1*, *Haplo2*, and *Haplo3. Haplo3* is present only in LAS39, suggesting that this variant involved in low antibody production was purged in relaxed line LAR16. This is reflected in the population differentiation between the lines as well, as LAS39 is most differentiated of the lines. The extent of differentiation at many markers suggests that these haplotypes were present as standing variants in the base population, as opposed to novel variants emerging in the LAS lineage after generation 24 and the founding of the relaxed line. Although we have no information for earlier generations, we can speculate on the driving forces acting on *Haplo3*. Given the complete purging of this haplotype from the relaxed lines, it seems reasonable to assume that this haplotype has a fitness cost; a fitness cost that may not have been evident in the base population or present as a cryptic variant. In the context of the LAS line, selection for *Haplo3* progressed slowly (no fixation after 39 generations), for which this inferred fitness cost is a possible explanation. As *Haplo3* is expected to segregate at an intermediate frequency at generation 24, this fitness cost would purge *Haplo3* quickly from the LAR line once selection is relaxed.

We observed only three synonymous SNP sites in the coding region of *SEMA5A*, with the majority of sequence polymorphism between haplotypes contained in intergenic and intronic regions. Taken together, this implies that the involvement of *SEMA5A* to the differential antibody response in the Virginia Antibody lines would be due to regulatory polymorphisms where the two haplotypes produce different expression levels of the *SEMA5A* protein, from different promoter or enhancer sequences. This could impact the expression of cytokines within this regulatory network and thus influence an effective and specific immune response.

### Transforming growth factor beta receptor 2

Earlier work with the Virginia body-weight selected lines, which have undergone a similar long-term experimental selection regime, has shown that the length of a selective-sweep is correlated with its contribution to the adaptive phenotype [10]. Hence, the individual sweep in our study that is most likely to make a significant contribution to antibody response is the long 8.7 Mb sweep region identified on GGA2. In this region, only one gene had GO immune terms, the transforming growth factor beta receptor 2 (*TGFBR2*), making it a logical candidate gene for further study. *TGFBR2* is a member of the serine/threonine protein kinase family, encoding a transmembrane protein that binds the secreted protein TGF-beta. The TGF signaling pathway regulates diverse biological processes during all stages of life and plays a key role in growth and the immune response [43, 44].

We observed fixation for different *TGFBR2* haplotypes in the selected lines. In-depth analyses comparing the sequence data for HAS39 and LAS39 suggest that these haplotypes differ by a 3712 bp deletion (between GGA2: 40,414,509 - 40,418,21) fixed in LAS39, which encompasses exons 4 and 5 of the *TGFBR2* gene prediction. Fixation of these haplotypes in the respective relaxed lines implies that the selected lines were already fixed (or nearly fixed) by generation 24, when the relaxed lines were founded. The length of this sweep, together with the rapid fixation, suggests that the selection acted strongly and fixation occurred swiftly, before multiple recombination events could break down the linkage within this region and leave a shorter sweep.

The strong selection signature suggests that the *TGFBR2* haplotypes might have played a major role in determining antibody response, most likely due to its key function in the TGF-beta pathway. Evidence is lacking, however, that this large sweep region was associated with 5 day antibody titers in the F_2_ intercross between HAS32 and LAS32, despite being covered by 7 segregating SNP markers (6 markers with MAF > 0.05; 605,124 bp or greater distance from the *TGFBR2* annotated gene). It should be noted, however, that these SNP markers were fixed or near fixed for alternative alleles in generation 39. As the population used for association analysis was small, the power in this analysis was low. Therefore it difficult to make strong conclusions as to whether these haplotypes had no or only minor effects on antibody response, or whether their functional significance was reliant on the genetic background of the population.

Defects in *TGFBR2* can result in serious debilitating and fatal diseases in humans, most notably the autosomal dominant aortic aneurysm syndrome, Loeys-Dietz syndrome, as well as an increased risk of certain cancers [45, 46]. That none of these conditions has been observed in LAS suggests that the deletion observed in LAS39 and LAR16 did not completely disrupt the function of the gene or that the function of this gene is less important in chickens than in humans. It is relevant to point out that mortality in these populations is low under our husbandry conditions. The deletion of only one set of duplicated exons may indicate that the LAS haplotype may still result in a functional *TGFBR2* protein, but with less splice variant options than the HAS haplotype.

### Major histocompatibility complex region

The major histocompatibility complex (*MHC*) is the most well studied immune gene complex in vertebrates due to its key role in the pathogen surveillance [47]. Accordingly the *MHC* has previously been investigated in the Virginia Antibody lines, albeit not at a sequence level [28, 29, 48]. The chicken *MHC* region is relatively small, simple and tightly-linked, compared to that of mammals, and thus termed the minimal essential *MHC* [49, 50]. Bidirectional selection on the *MHC* was evident as early as generation 12 in the Virginia Antibody lines, and alternative haplotypes were fixed by generation 32 (haplotype frequencies are summarized in Table 7; [28, 48]). Fixation for *B* ^*21*^ in HAS39 and *B* ^*13*^ in LAS39 was confirmed in our pooled genome sequencing and this divergent fixation contributed to the sweep signal on GGA16 from 2000 to 323,000 bp.

The sweep overlapping the *MHC* region spans a total 321 kb, and encompasses genes vital to antigen processing and presentation including *MHC* class I and II, TAP genes and tapasin. Association analysis between *MHC* haplotype and antibody response has demonstrated that the *B* ^*21*^ allele acts dominantly, with higher day 5 and day 12 titers of antibodies in homozygous and heterozygous *B* ^*21*^ individuals [48]. This explains the rapid fixation for the recessive *B* ^*13*^ in the LAS, as inferred by the fixation in LAR16, and why both haplotypes still segregate in HAR16. Although the HAS13 was close to fixation for *B* ^*21*^, at least one *B* ^*13*^ haplotype must have persisted until generation 24 the origin of the relaxed lines, as it continues to segregate in HAR16. The immunological advantage of the *B* ^*21*^ allele is well known in the context of Marek’s herpevirus [51]. Studies have also demonstrated that the *B* ^*21*^ haplotype has wider peptide recognition with more flexibility in the peptides it could bind, whereas *B* ^*13*^ is limited to peptides between 8 and 10 amino acids and negative anchor amino acids at certain sites [52–54].

## Conclusions

Adaptation to long-term, bidirectional selection for antibody response in this experimental chicken population has been facilitated by standing genetic variation across many regions of the genome. Although selective-sweep studies by themselves were unable to quantify the contribution by individual genes and polymorphisms to adaptation, data from earlier association and functional studies highlight three particularly interesting candidate sweeps and underlying candidate genes involved in immune surveillance (*MHC*) and immune regulation (*TGFBR2* and *SEMA5A*). Further work to investigate the large numbers of additional immune genes that are likely to have contributed to the response to selection in this experimental population would be useful to improve our understanding of selection on immune traits and to unravel the complexity of their interactions. These experimental lines continue to present a unique opportunity towards understanding the mode and tempo of selection in a higher vertebrate organism.

## Methods

### Experimental populations: the long-term bidirectionally selected Virginia Antibody chicken lines

The Virginia Antibody chicken lines were established from the Cornell randombred White Leghorn population [16]. From this base-population, two bidirectionally selected lines have been bred for high (HAS) and low (LAS) antibody response to an intravenous inoculation of 0.1 ml of 0.25% suspension SRBC, administered between 41 and 51 days of age. Plasma was collected five days after the inoculation and antibody response measured through a simple hemagglutination assay [55].

Selection has been carried out once every year, with approximately 120 chicks hatched per generation [16, 48]. Between generations 1–10, 7 males and 28 females were selected to produce the next generation of each line, and from generation 11 onwards, 8 males and 32 females were used. Within each line the parents for the next generation were selected from male and female groups of 30 and 60, respectively. Restricted truncation selection was used in each generation to avoid selection that would result in over representation of particular sire or dam families [48]. This procedure reduced, but could not avoid, inbreeding from common ancestry and did result in similar population structures in both lines.

Response to selection continues in HAS, whereas the LAS line appears to have reached a selection-plateau (Fig. 1). This plateau may be due to a threshold in response to the SRBC challenge or a limit in the technical sensitivity of the standard hemagglutination assay employed in antigen quantification [48, 56]. Relaxed sublines from both the HAS and LAS lines were established in generation 24. The relaxed lines from the high (HAR) and low (LAR) antibody response lines were founded by randomly selecting parents from the HAS and LAS lines, respectively.

DNA for the genomic analyses was prepared from blood samples collected from 16 to 30 individuals from each line and pooled in equimolar ratios prior to library construction (Table 8).

**Table 8:** Information about the populations, samples, phenotypes, and sequencing-depth used in the genomic analyses of the Virginia Antibody chicken lines

Population	*N*	Day 5 log_2_(AB titers)		Genome coverage
		Mean	sd	
HAS39	30	16.7	4.3	32.3
LAS39	30	2.6	1.6	36.7
HAR16	20	15.0	3.2	35.4
LAR16	16	5.3	2.2	34.8

Population: *H* High, *L* Low, *A* Antibody, *S* Selected, *R* Relaxed, 39-generation; *N* number of individuals in the sequenced pool; Genome Coverage: average sequence-depth of the pool; *AB* antibody

### Pooled whole-genome re-sequencing, sequence alignment, variant-calling and population genomic analyses

Genome sequencing library construction and sequencing was carried out by SciLifeLab (Uppsala, Sweden) using two lanes on an Illumina Hiseq 2500. Reads were aligned to the *Gallus gallus* genome (Galgal4; INSDC Assembly GCA_000002315.2, Nov 2011) using BWA [57]. Picard (v1.92; http://broadinstitute.github.io/picard/) was used to sort genomes and to mark and remove duplicates. GATK [58] was used for realignment around indels. Samtools (v3.3.0; [59, 60]) was used to generate mpileup files for population genomic comparisons. GATK UnifiedGenotyper was used to generate allele calls at all sites (option: emit all sites) and with ploidy = 30 to account for the pooled genome sample. Sites were filtered to only include those with >10 and <100 reads, wherefrom allele frequency, heterozygosity, and pairwise *F*_*ST*_ between all populations were calculated. PoPoolation2 (v1.1; [61]) was used to calculate *F*_*ST*_ over 1000 bp sliding windows with 50% overlaps between the population samples using the Karlsson et al. (v1.201; [62]) method, with minimum count 3, minimum coverage 10, maximum coverage 100, and minimum coverage fraction 1.

### Identification of candidate selective sweep regions

A stringent *F*_*ST*_ cutoff (>95% percentile *F*_*ST*_ = 0.946) was used when defining the sweeps to limit the number of candidate regions due to drift. Windows with *F*_*ST*_ values above this cutoff were clustered into candidate sweep regions when they were less than 0.5 Mb from one another (custom R scripts). Clusters containing only a single 1000 bp window, or less than 2 SNPs, were removed from the dataset.

### Estimating the expected contribution of genetic drift to the genomic divergence between the populations

Our experimental populations were small (*N*_*e*(1-10)_ = 22.4 and *N*_*e*(11-39)_ = 25.6; calculated *N*_*e*_ ~ 4*N_ef_*N_*em*_/(*N*_*ef*_ + *N*_*em*_)), whereby genetic drift could be a prominent force affecting allele frequencies which would confound the identification of selective sweep signatures. Simulations were carried out for a 5 Mb locus using SFS_CODE [20] taking into account mutation (the high mitochondrial mutation rate of 3.13 × 10-7 was implemented to promote high standing genetic variation in the ancestral population; [63]), recombination (6.4 cM/Mb for microchromosomes and 720 2.8 cM/Mb for macrochromosomes; [64]) change in population sizes between ancestral breed to the selected lines (effective population size of 500 was chosen to allow for generation of mutations and rearrangement of haplotypes in the base population then population size reduced to 25 for 39 generations for selected lines) and proportion of females (0.8) in the selective breeding scheme.

Resulting allele frequencies from simulations were used to calculate *F*_*ST*_ between the two simulated populations. Average *F*_*ST*_ was calculated per 1000 bp window with 50% overlaps then regions of differentiation were clustered together if distance between differentiated SNP sites was less than 50 kb, following a similar methodology applied to the real genomic data.

### Gene ontology analysis to identify immune genes within differentiated regions

We used GO analysis to identify functional candidate sweeps that were enriched for immune-related genes. BEDOPS [65] was used to extract all Ensembl geneIDs underlying candidate sweep regions then used for GO annotation analysis via i) the Database for Annotation, Visualization and Integrated Discovery [66] and ii) the Protein ANalysis THrough Evolutionary Relationships PANTHER v10.0; [67, 68]). GeneIDs with immunological GO terms were identified and mapped back onto candidate sweep clusters.

To confirm transcription of the genes predicted by Ensembl genebuild within candidate sweep regions, White Leghorn spleen RNA sequence reads were accessed from the Short Read Archive (http://www.ncbi.nlm.nih.gov/sra; runs SRR2889287, SRR2889288, SRR2889289) and aligned using STAR (v2.3.1; [69]). Genome coverage of the RNAseq alignments was calculated with BEDTools v2.25.0; [70]. RNAseq assemblies and genome coverage were visualized in IGV (v2.3.52; [71, 72]).

### Identification of associations between candidate selective sweep regions and immune-traits

A number of QTL and GWAS studies have been performed for immunological traits in chickens, including an analysis of an F_2_ intercross between the HAS and LAS populations. Data and results from these studies were used to increase our confidence in individual candidate sweep regions. We reanalyzed the SNP association data from Dorshorst et al. [48] to compare the overlap between associations in those data and our candidate selective sweeps using a multi-locus, adaptive backward-elimination model-selection approach [10]. Briefly, this dataset consisted of 128 individuals representing the phenotypic extremes in 5-day antibody titer sampled from an F_2_ intercross generated by intercrossing birds from HAS32 and LAS32 that were genotyped by a custom GoldenGate® Genotyping assay containing 3072 SNP markers as described by Dorshorst et al. [48].

In order to focus on the most informative markers that tag divergent selective sweep regions, we used a subset of SNP markers that possessed an allele frequency difference > 0.7 in the pooled genome sequence between HAS39 and LAS39. To further refine this subset, neighboring, linked markers were clustered together (< 5 Mb between SNPs in a cluster, > 5 Mb between clusters). Backward elimination was applied to clusters with more than one marker to select the most significant marker as representative of the cluster region for use in the genome-wide analysis. This final refined SNP subset was analyzed using the same backward elimination process as in the within-cluster analysis. All analyses used a standard linear model framework, starting with a full model including the fixed effects of sex and the additive effects of the highly differentiated markers. These were regressed onto log_2_ transformed day 5 antibody titers, and the final model from the backward elimination analysis was decided using an adaptive 5–20% FDR criterion [73, 74].

### Estimating haplotype segregation within candidate sweep regions

We do not have DNA and thus genomes from the line founders, so software estimating haplotype frequencies within pooled sequencing samples [75] cannot be applied. However, this experimental population has a well-documented population history and extreme genomic differentiation within the defined candidate sweep regions, affording us the opportunity to disentangle highly divergent haplotypes based on allele frequency differences observed in the sequencing data. Where both lines are fixed for different haplotypes, these regions can be identified by homozygosity and haplotypes can be determined by adjusting all allele frequencies to the reference haplotype from one line. In other cases, one line is fixed within a sweep region, while different haplotypes continue to segregate in the other line. Here, positions are sequentially filtered on an ad hoc basis by allele frequencies differing from the selected reference haplotype present in the line fixed for one haplotype. Allele frequencies in all lines are adjusted to reflect alternate allele frequencies from the reference haplotype, allowing visualization and inference of alternative segregating haplotypes (see also Additional file 7). As shown in the results, this approach was useful to estimate haplotype-frequencies at candidate genes.

## Additional files

Additional file 1: Summary information for candidate sweep regions. (XLSX 68 kb)
Additional file 2: List of GO Biological Process enrichment. (XLSX 12 kb)
Additional file 3: List of PANTHER Overrepresentation Test results. (XLSX 21 kb)
Additional file 4: List of SNP marker names and genome locations. (TXT 3 kb)
Additional file 5: Candidate sweep region in relation to rs14207559 and *SEMA5A* gene. (PDF 2121 kb)
Additional file 6: Coverage in region of the TGFBR2 gene demonstrating deletion observed in LAS39 and LAR16. (PDF 211 kb)
Additional file 7: Diagram illustrating method for inferring haplotype frequencies within sweep regions. (PDF 352 kb)

## References

[1] Chevin LM, Hospital F (2008). Selective sweep at a quantitative trait locus in the presence of background genetic variation. Genetics.

[2] Pritchard JK, Pickrell JK, Coop G (2010). The genetics of human adaptation: hard sweeps, soft sweeps, and polygenic adaptation. Curr Biol.

[3] Jensen JD (2014). On the unfounded enthusiasm for soft selective sweeps. Nat Commun.

[4] Elena SF, Lenski RE (2003). Evolution experiments with microorganisms: the dynamics and genetic bases of adaptation. Nat Rev Genet.

[5] Barrick JE, Yu DS, Yoon SH, Jeong H, Oh TK, Schneider D, Lenski RE, Kim JF (2009). Genome evolution and adaptation in a long-term experiment with Escherichia coli. Nature.

[6] Burke MK (2012). How does adaptation sweep through the genome? Insights from long-term selection experiments. Proc R Soc B Biol Sci.

[7] Hermisson J, Pennings PS (2005). Soft sweeps: molecular population genetics of adaptation from standing genetic variation. Genetics.

[8] Hernandez RD, Kelley JL, Elyashiv E, Melton SC, Auton A, McVean G, Sella G, Przeworski M, Genomes P (2011). Classic selective sweeps were rare in recent human evolution. Science.

[9] Burke MK, Liti G, Long AD (2014). Standing genetic variation drives repeatable experimental evolution in outcrossing populations of Saccharomyces cerevisiae. Mol Biol Evol.

[10] Sheng ZY, Pettersson ME, Honaker CF, Siegel PB, Carlborg O (2015). Standing genetic variation as a major contributor to adaptation in the Virginia chicken lines selection experiment. Genome Biol.

[11] Pritchard JK, Di Rienzo A (2010). Adaptation - not by sweeps alone. Nat Rev Genet.

[12] Remolina SC, Chang PL, Leips J, Nuzhdin SV, Hughes KA (2012). Genomic basis of aging and life-history evolution in *Drosophila melanogaster*. Evolution.

[13] Turner TL, Miller PM, Cochrane VA (2013). Combining genome-wide methods to investigate the genetic complexity of courtship song variation in drosophila melanogaster. Mol Biol Evol.

[14] Jalvingh KM, Chang PL, Nuzhdin SV, Wertheim B (2014). Genomic changes under rapid evolution: selection for parasitoid resistance. Proc R Soc B Biol Sci.

[15] Martins NE, Faria VG, Nolte V, Scholtterer C, Teixeira L, Sucena E, Magalhaes S (2014). Host adaptation to viruses relies on few genes with different cross-resistance properties. Proc Natl Acad Sci U S A.

[16] Siegel PB, Gross WB (1980). Production and persistence of antibodies in chickens to sheep erythrocytes.1. Directional selection. Poult Sci.

[17] Siegel PB, Gross WB, Cherry JA (1982). Correlated responses of chickens to selection for production of antibodies to sheep erythrocytes. Anim Blood Groups Biochem Genet.

[18] Boa-Amponsem K, Dunnington EA, Siegel PB (1997). Antibody transmitting ability of hens from lines of chickens differing in response to SRBC antigen. Br Poultry Sci.

[19] Johansson AM, Pettersson ME, Siegel PB, Carlborg O (2010). Genome-wide effects of long-term divergent selection. PLoS Genet.

[20] Hernandez RD (2008). A flexible forward simulator for populations subject to selection and demography. Bioinformatics.

[21] Groenen MAM, Wahlberg P, Foglio M, Cheng HH, Megens HJ, Crooijmans R, Besnier F, Lathrop M, Muir WM, Wong GKS (2009). A high-density SNP-based linkage map of the chicken genome reveals sequence features correlated with recombination rate. Genome Res.

[22] Zhou H, Li H, Lamont SJ (2003). Genetic markers associated with antibody response kinetics in adult chickens. Poult Sci.

[23] Siwek M, Cornelissen SJB, Nieuwland MGB, Buitenhuis AJ, Bovenhuis H, Crooijmans R, Groenen MAM, Vries-Reilingh G, Parmentier HK, van der Poel JJ (2003). Detection of QTL for immune response to sheep red blood cells in laying hens. Anim Genet.

[24] Siwek M, Buitenhuis B, Cornelissen S, Nieuwland M, Knol EF, Crooijmans R, Groenen M, Parmentier H, van der Poel J (2006). Detection of QTL for innate: Non-specific antibody levels binding LPS and LTA in two independent populations of laying hens. Dev Comp Immunol.

[25] Biscarini F, Bovenhuis H, van Arendonk JAM, Parmentier HK, Jungerius AP, van der Poel JJ (2010). Across-line SNP association study of innate and adaptive immune response in laying hens. Anim Genet.

[26] Zhang L, Li P, Liu RR, Zheng MQ, Sun Y, Wu D, Hu YD, Wen J, Zhao GP (2015). The identification of loci for immune traits in chickens using a genome-wide association study. PLoS One.

[27] Geng TY, Guan XJ, Smith EJ (2015). Screening for genes involved in antibody response to sheep red blood cells in the chicken, Gallus gallus. Poult Sci.

[28] Dunnington EA, Martin A, Briles RW, Briles WE, Gross WB, Siegel PB (1989). Antibody-responses to sheep erythrocytes for White Leghorn chickens differing in haplotypes of the Major Histocompatibility Complex (B). Anim Genet.

[29] Martin A, Dunnington EA, Gross WB, Briles WE, Briles RW, Siegel PB (1990). Production traits and alloantigen systems in lines of chickens selected for high or low antibody-responses to sheep erythrocytes. Poult Sci.

[30] Dunnington EA, Siegel PB (1996). Long-term divergent selection for eight-week body weight in White Plymouth Rock chickens. Poult Sci.

[31] Hill WG (2010). Understanding and using quantitative genetic variation. Philos Trans R Soc B Biol Sci.

[32] Burke MK, Dunham JP, Shahrestani P, Thornton KR, Rose MR, Long AD (2010). Genome-wide analysis of a long-term evolution experiment with Drosophila. Nature.

[33] Tobler R, Franssen SU, Kofler R, Orozco-terWengel P, Nolte V, Hermisson J, Schlotterer C (2014). Massive habitat-specific genomic response in D. melanogaster populations during experimental evolution in hot and cold environments. Mol Biol Evol.

[34] Coop G, Pickrell JK, Novembre J, Kudaravalli S, Li J, Absher D, Myers RM, Cavalli-Sforza LL, Feldman MW, Pritchard JK (2009). The role of geography in human adaptation. PLoS Genet.

[35] Janeway CAJ, Travers P, Walport M (2001). Immunobiology: the immune system in health and disease.

[36] Gross WG, Siegel PB, Hall RW, Domermuth CH, Duboise RT (1980). Production and persistence of antibodies in chickens to sheep erythrocytes.2. Resistance to infectious-diseases. Poult Sci.

[37] Bumstead N (1998). Genetic resistance to avian viruses. Revue Scientifique et Technique de l’Office International des Epizooties.

[38] Koenen ME, Boonstra-Blom AG, Jeurissen SHM (2002). Immunological differences between layer- and broiler-type chickens. Vet Immunol Immunopathol.

[39] Kramer J, Visscher AH, Wagenaar JA, Cornelissen J, Jeurissen SHM (2003). Comparison of natural resistance in seven genetic groups of meat-type chicken. Br Poultry Sci.

[40] Lyu MG, Li Y, Hao YT, Sun TT, Liu WJ, Lyu CC, Fu RF, Li HY, Xue F, Liu XF (2015). Elevated Semaphorin 5A correlated with Th1 polarization in patients with chronic immune thrombocytopenia. Thromb Res.

[41] Sugimoto M, Fujikawa A, Womack JE, Sugimoto Y (2006). Evidence that bovine forebrain embryonic zinc finger-like gene influences immune response associated with mastitis resistance. Proc Natl Acad Sci U S A.

[42] Melin M, Carlsson B, Anckarsater H, Rastam M, Betancur C, Isaksson A, Gillberg C, Dahl N (2006). Constitutional downregulation of SEMA5A expression in autism. Neuropsychobiology.

[43] Letterio JJ, Roberts AB (1998). Regulation of immune responses by TGF-beta. Annu Rev Immunol.

[44] Li MO, Wan YY, Sanjabi S, Robertson AKL, Flavell RA (2006). Transforming growth factor-beta regulation of immune responses. Annual Review of Immunology.

[45] Loeys BL, Schwarze U, Holm T, Callewaert BL, Thomas GH, Pannu H, De Backer JF, Oswald GL, Symoens S, Manouvrier S (2006). Aneurysm syndromes caused by mutations in the TGF-beta receptor. N Engl J Med.

[46] Levy L, Hill CS (2006). Alterations in components of the TGF-beta superfamily signaling pathways in human cancer. Cytokine Growth Factor Rev.

[47] Klein J (1986). Natural history of the major histocompatibility complex.

[48] Dorshorst BJ, Siegel PB, Ashwell CM (2011). Genomic regions associated with antibody response to sheep red blood cells in the chicken. Anim Genet.

[49] Kaufman J, Milne S, Gobel TWF, Walker BA, Jacob JP, Auffray C, Zoorob R, Beck S (1999). The chicken B locus is a minimal essential major histocompatibility complex. Nature.

[50] Kaufman J (2015). What chickens would tell you about the evolution of antigen processing and presentation. Curr Opin Immunol.

[51] Briles WE, Stone HA, Cole RK (1977). Mareks-disease - effects of B-histocompatibility alloalleles in resistant and susceptible chicken lines. Science.

[52] Sherman MA, Goto RM, Moore RE, Hunt HD, Lee TD, Miller MM (2008). Mass spectral data for 64 eluted peptides and structural modeling define peptide binding preferences for class I alleles in two chicken MHC-B haplotypes associated with opposite responses to Marek's disease. Immunogenetics.

[53] Hedrick PW (1998). Balancing selection and MHC. Genetica.

[54] Charbonnel N, Pemberton J (2005). A long-term genetic survey of an ungulate population reveals balancing selection acting on MHC through spatial and temporal fluctuations in selection. Heredity.

[55] Wegmann TG, Smithies O (1966). A simple hemagglutination system requiring small amounts of red cells and antibodies. Transfusion.

[56] Zhao XL, Honaker CF, Siegel PB (2012). Phenotypic responses of chickens to long-term selection for high or low antibody titers to sheep red blood cells. Poult Sci.

[57] Li H, Durbin R (2009). Fast and accurate short read alignment with Burrows-Wheeler transform. Bioinformatics.

[58] McKenna A, Hanna M, Banks E, Sivachenko A, Cibulskis K, Kernytsky A, Garimella K, Altshuler D, Gabriel S, Daly M (2010). The genome analysis toolkit: a MapReduce framework for analyzing next-generation DNA sequencing data. Genome Res.

[59] Li H, Handsaker B, Wysoker A, Fennell T, Ruan J, Homer N, Marth G, Abecasis G, Durbin R (2009). Genome project data P: the sequence alignment/map format and SAMtools. Bioinformatics.

[60] Li H (2011). A statistical framework for SNP calling, mutation discovery, association mapping and population genetical parameter estimation from sequencing data. Bioinformatics.

[61] Kofler R, Orozco-terWengel P, De Maio N, Pandey RV, Nolte V, Futschik A, Kosiol C, Schloetterer C (2011). PoPoolation: a toolbox for population genetic analysis of next generation sequencing data from pooled individuals. Plos One.

[62] Karlsson EK, Baranowska I, Wade CM, Salmon Hillbertz NHC, Zody MC, Anderson N, Biagi TM, Patterson N, Pielberg GR, Kulbokas EJ (2007). Efficient mapping of mendelian traits in dogs through genome-wide association. Nat Genet.

[63] Alexander M, Ho SYW, Molak M, Barnett R, Carlborg O, Dorshorst B, Honaker C, Besnier F, Wahlberg P, Dobney K (2015). Mitogenomic analysis of a 50-generation chicken pedigree reveals a rapid rate of mitochondrial evolution and evidence for paternal mtDNA inheritance. Biol Lett.

[64] Hillier LW, Miller W, Birney E, Warren W, Hardison RC, Ponting CP, Bork P, Burt DW, Groenen MAM, Delany ME (2004). Sequence and comparative analysis of the chicken genome provide unique perspectives on vertebrate evolution. Nature.

[65] Neph S, Kuehn MS, Reynolds AP, Haugen E, Thurman RE, Johnson AK, Rynes E, Maurano MT, Vierstra J, Thomas S (2012). BEDOPS: high-performance genomic feature operations. Bioinformatics.

[66] Huang DW, Sherman BT, Lempicki RA (2009). Systematic and integrative analysis of large gene lists using DAVID bioinformatics resources. Nat Protoc.

[67] Thomas PD, Campbell MJ, Kejariwal A, Mi HY, Karlak B, Daverman R, Diemer K, Muruganujan A, Narechania A (2003). PANTHER: a library of protein families and subfamilies indexed by function. Genome Res.

[68] Mi H, Poudel S, Muruganujan A, Casagrande JT, Thomas PD (2016). PANTHER version 10: expanded protein families and functions, and analysis tools. Nucleic Acids Res.

[69] Dobin A, Davis CA, Schlesinger F, Drenkow J, Zaleski C, Jha S, Batut P, Chaisson M, Gingeras TR (2013). STAR: ultrafast universal RNA-seq aligner. Bioinformatics.

[70] Quinlan AR, Hall IM (2010). BEDTools: a flexible suite of utilities for comparing genomic features. Bioinformatics.

[71] Robinson JT, Thorvaldsdottir H, Winckler W, Guttman M, Lander ES, Getz G, Mesirov JP (2011). Integrative genomics viewer. Nat Biotechnol.

[72] Thorvaldsdottir H, Robinson JT, Mesirov JP (2013). Integrative Genomics Viewer (IGV): high-performance genomics data visualization and exploration. Brief Bioinform.

[73] Abramovich F, Benjamini Y, Donoho DL, Johnstone IM (2006). Adapting to unknown sparsity by controlling the false discovery rate. Ann Stat.

[74] Gavrilov Y, Benjamini Y, Sarkar SK (2009). An adaptive step-down procedure with proven fdr control under independence. Ann Stat.

[75] Kessner D, Turner TL, Novembre J. Maximum Likelihood Estimation of Frequencies of Known Haplotypes from Pooled Sequence Data. Mol Biol Evol. 2013;30:5:1145–58. 10.1093/molbev/mst016 PMC3670732 23364324

