# Supplementary material for: Genome-wide standing variation facilitates long-term response to bidirectional selection for antibody response in chickens
Source: BMC Genomics. 2017 Jan 18;18:99. doi: 10.1186/s12864-016-3414-7 (PMC5244587; doi:10.1186/s12864-016-3414-7)
Supplement: Additional file 5: — Candidate sweep region in relation to rs14207559 and SEMA5A gene. (PDF 2121 kb) [file 12864_2016_3414_MOESM5_ESM.pdf]

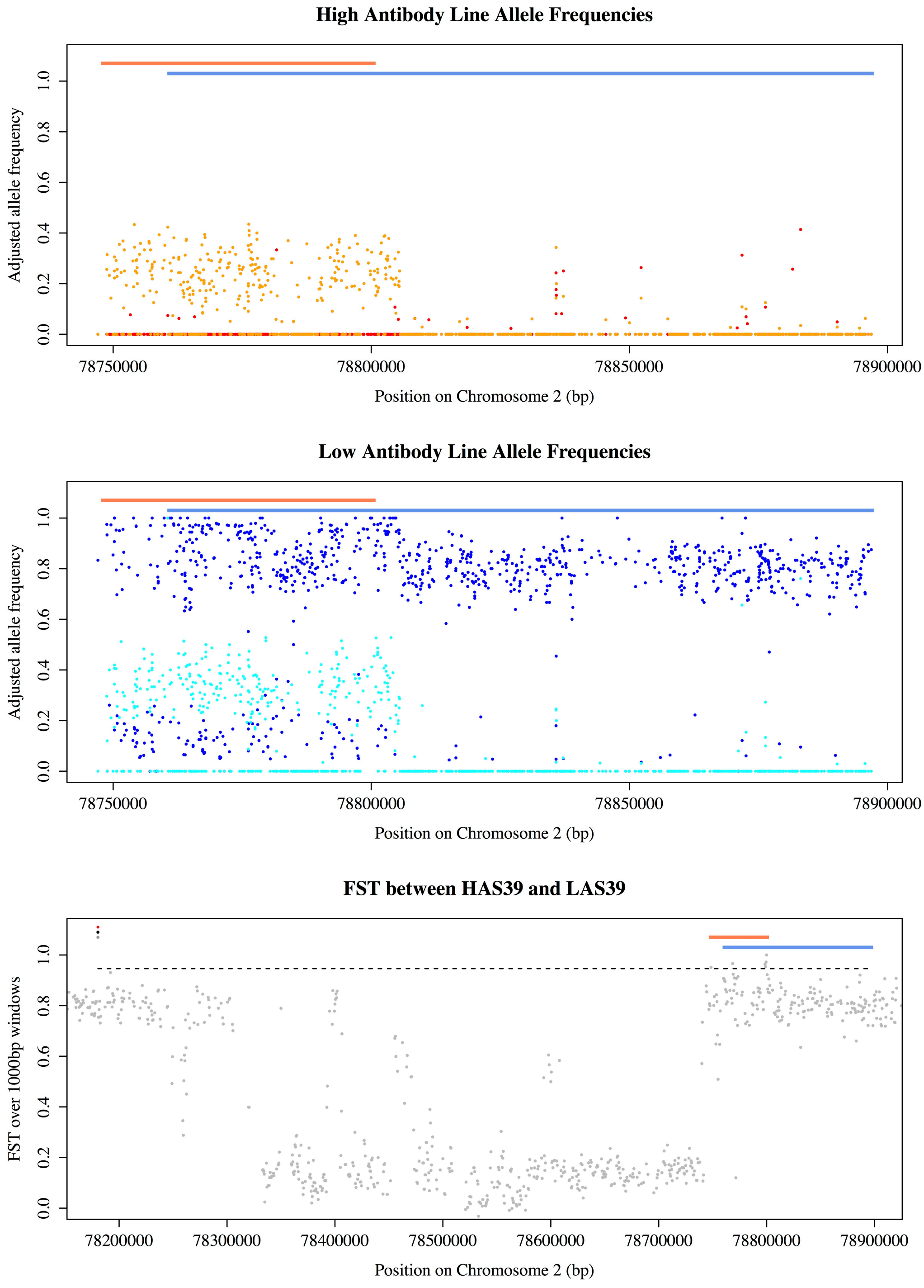

Additional File 5 (figure): Candidate sweep region in relation to rs14207559 and *SEMA5A* gene, showing allele frequency in HAS39 (red) and HAR16 (orange) (upper figure), LAS39 (blue) and LAR16 (teal) (middle figure) and the FST between HAS39 and LAS39 (grey) across a wider genomic region to show location of SNP marker rs14207559 (red dot) (lower figure). Candidate sweep region indicated with orange line and SEMA5A gene indicated with blue line in all figures.
