# Supplementary material for: Genome-wide standing variation facilitates long-term response to bidirectional selection for antibody response in chickens
Source: BMC Genomics. 2017 Jan 18;18:99. doi: 10.1186/s12864-016-3414-7 (PMC5244587; doi:10.1186/s12864-016-3414-7)
Supplement: Additional file 6: — Coverage in region of the TGFBR2 gene demonstrating deletion observed in LAS39 and LAR16. (PDF 211 kb) [file 12864_2016_3414_MOESM6_ESM.pdf]

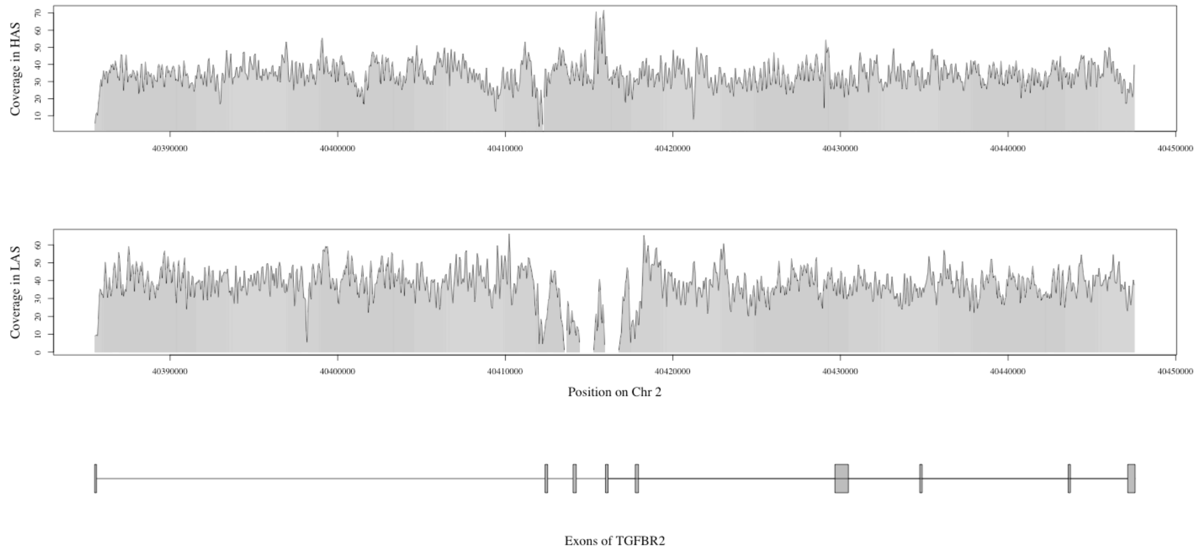

Additional File 6 (figure): Coverage in region (40,413,000-40,419,000) of the TGFBR2 gene (chr 2: 40,385,525 - 40,447,574) demonstrating deletion observes in LAS39 and LAR16, indicating fixation for the deletion-haplotype in the LAS line prior to founding of the relaxed line.
