# Supplementary material for: Genome-wide standing variation facilitates long-term response to bidirectional selection for antibody response in chickens
Source: BMC Genomics. 2017 Jan 18;18:99. doi: 10.1186/s12864-016-3414-7 (PMC5244587; doi:10.1186/s12864-016-3414-7)
Supplement: Additional file 7: — Diagram illustrating method for inferring haplotype frequencies within sweep regions. (PDF 352 kb) [file 12864_2016_3414_MOESM7_ESM.pdf]

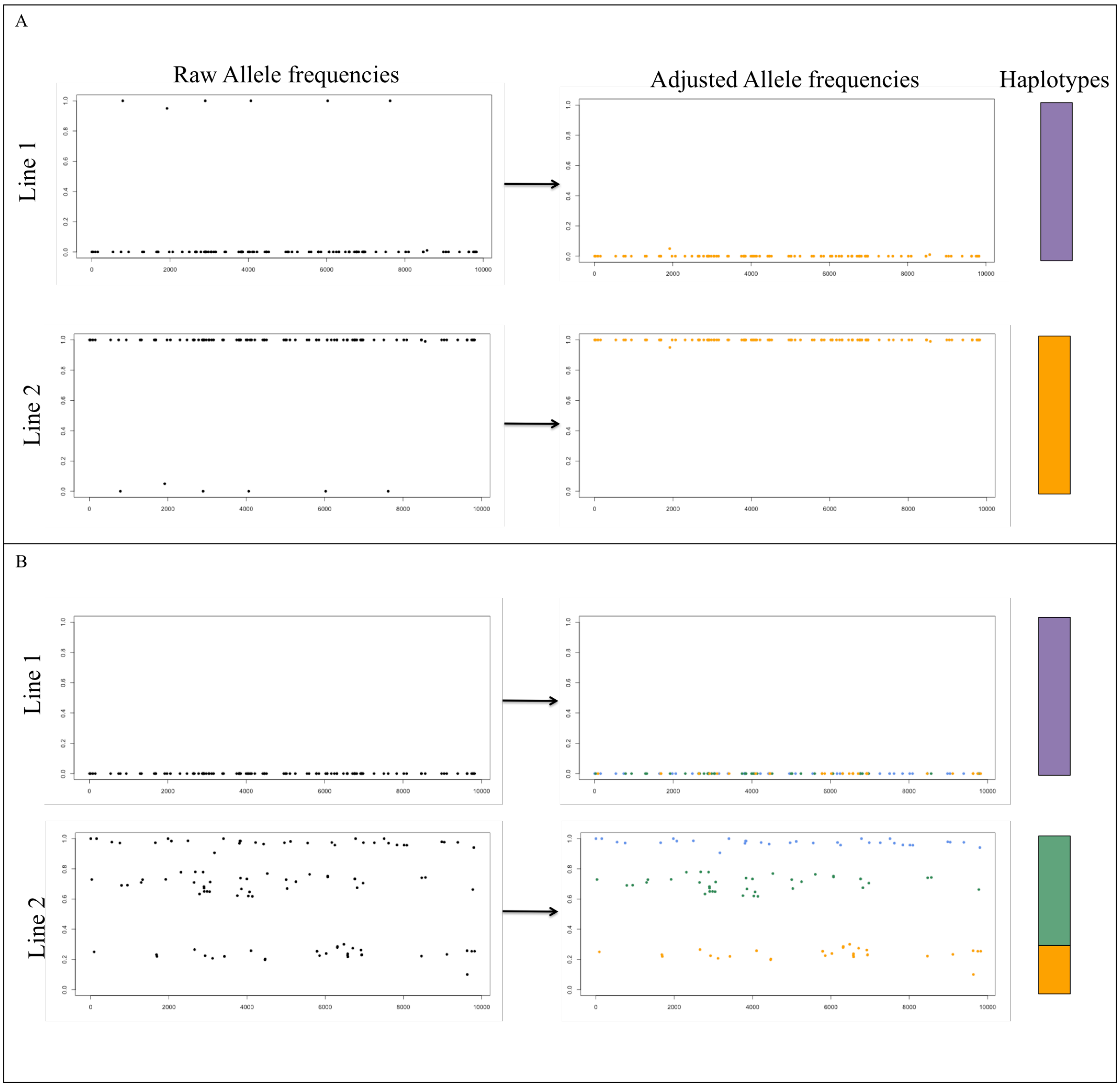

Additional File 7 (figure): Method for inferring haplotype frequencies within sweep regions in cases where the two lines are fixed for different haplotypes (A) and where one line is fixed for one haplotype and the other line has two different haplotypes still segregating (B).
A. Raw allele frequencies are present in black (left); adjusted allele frequencies, calculated as the alternate allele frequency to the reference halplotype represented in orange (middle); inferred major haplotypes represented in purple and orange bars (right)
B. Raw allele frequencies are present in black (left); adjusted allele frequencies, calculated as the alternate allele frequency to the reference halplotype represented in three colours (middle), where orange represents the positions contributing to one haplotype, the green positions contributing the the other haplotype, and blue are positions on both haplotypes such that allele frequency approximately =1; inferred haplotype represented in purple and orange (right).
